# Optimal Appearance Model for Visual Tracking

**DOI:** 10.1371/journal.pone.0146763

**Published:** 2016-01-20

**Authors:** Yuru Wang, Longkui Jiang, Qiaoyuan Liu, Minghao Yin

**Affiliations:** 1 Computer Science and Information Technology, North-East Normal University, Changchun, Jilin Province, China; 2 School of Information Engineering, Jilin Business and Technology College, Changchun, Jilin Province, China; University of Verona, ITALY

## Abstract

Many studies argue that integrating multiple cues in an adaptive way increases tracking performance. However, what is the definition of adaptiveness and how to realize it remains an open issue. On the premise that the model with optimal discriminative ability is also optimal for tracking the target, this work realizes adaptiveness and robustness through the optimization of multi-cue integration models. Specifically, based on prior knowledge and current observation, a set of discrete samples are generated to approximate the foreground and background distribution. With the goal of optimizing the classification margin, an objective function is defined, and the appearance model is optimized by introducing optimization algorithms. The proposed optimized appearance model framework is embedded into a particle filter for a field test, and it is demonstrated to be robust against various kinds of complex tracking conditions. This model is general and can be easily extended to other parameterized multi-cue models.

## Introduction

The goal of visual tracking is to obtain the state of interest target including the location and motion data. Many efficient tracking methods developed in the past three decades demonstrate the importance of modeling the appearance of a target. In summary, it essentially determines the robustness and stability of tracking systems. Tracking performance depends primarily on how discriminative the appearance model is in distinguishing an object from its surroundings.

The main challenges in constructing appearance models are the following: (i) The complexity of background: The essential problem of tracking is to find the classification margin between the target object and its background. In most tracking problems, the scene is very complex and contains illumination changes, similar objects, partial occlusion, abrupt scene changes, etc.; these factors make it difficult to find a good margin that allows for a clear classification between the two classes. (ii) The complexity and variety of the target’s appearance: Targets, especially non-rigid targets, always change their shape and show complex inner structural deformation, which challenges appearance modeling methods. Despite extensive research, this method still suffers from difficulties in handling complex tracking conditions [1].

Many appearance models are well-designed for describing targets, including color [2], texture [2], motion [3], sparse coding method [4], etc. However, the models based on a single feature failed to provide a discriminative description for some complex tracking conditions. Therefore, most researchers focus on multi-cue integration models. To the extent that is possible, the cues employed in multi-cue trackers must be orthogonal to each other, so that they are able to cooperate in providing robust and stable representations [5]. Orthogonal cues are possible in patch-based models [6] [7], whereby a single feature is employed in modeling different parts of the target. Although powerful patch-based models have been proposed, prior knowledge is necessary; more importantly, the target size must be large enough to be represented in sections. Although a partition is realized, describing their combination structure is still a problem. An alternative method is to represent the target as an integration of different visual cues. Much effort has been made to develop such models. In Birchfield’s early studies [8], an elliptical head tracker was developed that performed a local search employing image gradients and a color histogram model. His work offered a preliminary combination of the visual model; however, the model was not robust enough, because less consideration was given to tracking conditions. In addition, cascade models [9] [10] integrated multiple cues in a hierarchical way. This kind of model works with the advantage of less complexity, but the tracking accuracy is not greatly improved; moreover, the sequence in the cascade is poses additional problems.

In real tracking conditions, different visual features have different discriminative ability. If they are assigned equal importance-regardless of the combination way that is employed-the model will have low robustness. Therefore, the parameters for the multi-cue integration model should be adaptive to the changes in tracking conditions. To address this problem, Triesch and Malsburg [11] introduced the concept of “adaptiveness” into the visual model and proposed a dynamic framework to adaptively integrate different cues. In their democratic integration framework, each cue contributes to the joint result according to its reliability. Following such a strategy, a number of studies, e.g. [12] [13] [14] proposed adaptive multi-cue integration models and improved the tracking accuracy. For example, P*é*rez [13] realized adaptiveness by updating the model with the reliability of specific cues in the previous frame. Brasnett [14] made an improvement to P*é*rez’s model and added the measurement of the current frame in evaluating the cue’s importance.

The concept of “adaptiveness” in the so-called multi-cue integration adaptive model is to adapt the importance of each feature to the change in tracking conditions. If the employed cues are orthogonal, the key problem is to place greater confidence in the features with stronger performance and less confidence in those with weaker performance. The crucial point then becomes one of evaluating the discriminative ability for a specific feature. To address this problem, Collins [15] proposed an online selection algorithm of discriminative tracking features-according to log likelihood ratios of class conditional sample densities from the object and background-to form a new set of candidate features tailored to the local object/background discrimination task. Wang [16] defined a feature evaluation method and implemented a tracking method to control the abrupt adaption. In addition, Khanloo [17] introduced a max-margin tracker to linearly combine the constant and adaptive appearance features. Similar studies include the reliability based fusing method [18]. However, how is the performance of the adaptive scheme evaluated? There must be a preferable way to integrate multiple cues. The concept of adaptiveness should fulfill the following rule: The model will give the best description of the target’s appearance that is robust to changeable tracking conditions. Furthermore, in the feature space, the projection of the pixels in the target and background regions will optimize the margin between two classes, to realize accurate tracking. Therefore, a key component of this work is to achieve optimization and adaptiveness in the multi-cue integration model.

When an appropriate multi-cue integration model is defined, it is necessary to optimize the parameters of the model at each time step according to the change in tracking conditions, to give an optimal representation of a target’s current appearance. This issue referred to as a global optimization problem; the objective function is “optimal”, and the solution to the problem is the parameters involved in the model. How does one adapt the optimized parameters and describe the “optimal”?

This paper transforms the modeling problem of adaptive appearance into a global single objective optimization issue. To give a description of “optimal”, a set of discrete samples are generated to approximate the distribution of pixels in the target and its surroundings. Drawing upon margin theory, we analyze the distribution of these samples, and define an objective function related to the classification margin. Then, to realize adaptiveness, we introduce optimization algorithms to optimize the model parameters. Specifically, the proposed adaptive model framework is embedded into a particle filter to perform a field test. Tests on videos with different complex appearances show its robustness.

Compared with previous approaches, our method starts with the analysis of adaptiveness and introduces the idea of optimization into model building for the first time. Furthermore, the proposed solution to the multiple cues integration model is suitable for most parameterized models and can be extended to various kinds of features and tracking methods.

The next section presents a brief look at the proposed appearance model optimization scheme. The section “Tracking” adapts these ideas to the task of target tracking and develops an online optimized adaptive model in a particle filter framework. In the last section, experiments are presented to illustrate how the method adapts to the changing appearance of both the tracked object and the scene background.

## Optimal appearance model

Our goal is to model the target’s appearance in an optimal way. Given a candidate feature set and integration model, we combine prior knowledge and current observation, and define an evaluation function of a visual model, realizing optimization by optimizing the model parameters. The proposed optimal appearance model is suitable for different feature sets and any parameterized integration model.

The following steps are taken. First, a set of samples are evolved from the most recently tracked frame. Second, an objective function to optimize the classification margin is defined based on the statistical analysis on the observed feature space. Finally, the model is optimized through the iterative parameter optimization step.

### Discrete samples

We hypothesize that the features that best discriminate the object and background are also best for tracking the target. At time t, an approximate state can be computed according to prior knowledge. If a sufficient discriminative model is employed to observe them, the pixels lying in the target and its background will show large similarity distance. However, we cannot obtain the target’s real state and cannot observe the target and its background directly in real problems; thus a Monte Carlo simulation method is employed to generate samples and approximate the distribution of foreground and background pixels. These samples are not generated randomly but are associated with the prior knowledge.

**Fig 1:**
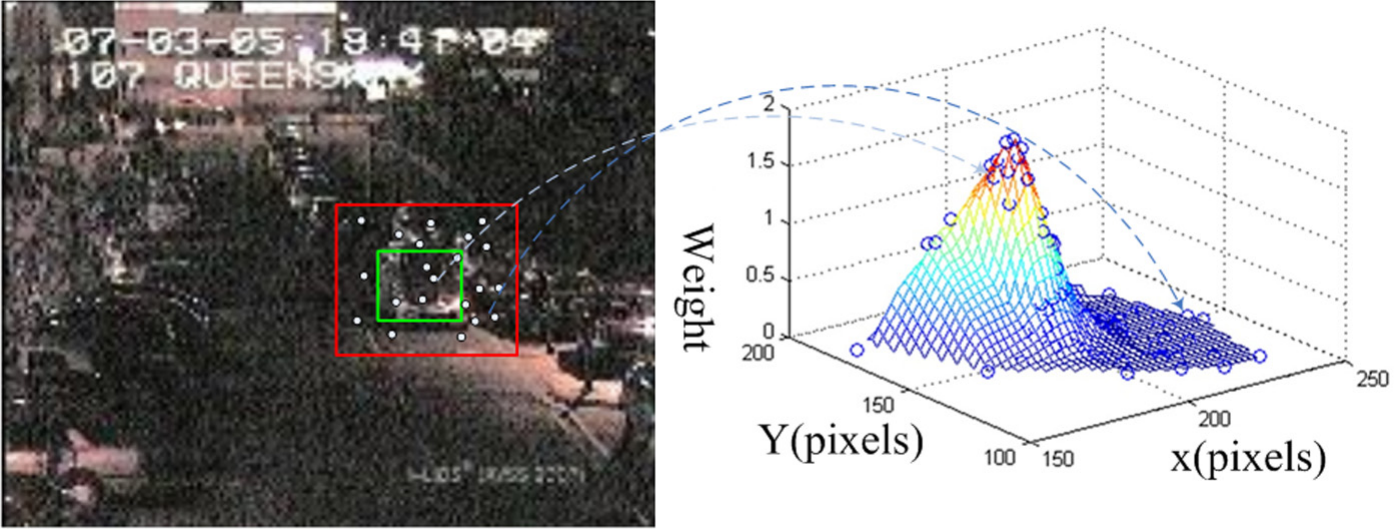
Samples are generated in the foreground (the green box of the left image) and background regions (the region between the red and green box), and if they are observed on a discriminative feature, the right figure gives their weights. The foreground samples are mapped to the peak region, while the background samples are projected to the low weight region.

We define a rectangular region covering the object for positive sampling and a larger surrounding rectangular ring for background sampling. As shown in Fig 1, an inner rectangle of dimension *h* × *w* pixels and an outer margin of width *γ* × max(*h*,*w*) pixels are located for generating samples. In addition *γ* is a parameter controlling the margin size. A prior knowledge-dependent method can be used to explicitly define the background region, for example, defining margins with unequal sizes for different directions by predicting the motion of the target. In our realization, more samples are generated in the predicted motion area.

Specifically, *n* samples are randomly generated from a Gaussian distribution in the first frame, and assigned with initial equal weights, as $S_{0}={\left\{\omega_{0}^{i},x_{0}^{i}\right\}}_{i=1}^{n}$.

$$x_{0}^{i}∼G\left(\mu_{0},\sigma_{0}\right)$$ (1)

where, *G* is a Gaussian distribution with average and variance values of *μ*_0_ and *σ*_0_.

At time *t*, the evolved *n* samples are employed for the adaptive model. With the prior knowledge of the target’s state ${\hat{X}}_{t-1}$ and the samples *S*_*t*−1_ employed at time *t*−1, new samples are generated according to the following formula:

$$S_{t}=S_{t,1}\cup S_{t,2}$$ (2)

where, *S*_*t*,1_ and *S*_*t*,2_ are two sample sets generated individually, and they cooperate to generate *n* new samples. Samples in *S*_*t*,1_ are evolved from *S*_*t*−1_.

$$x_{t}^{i}∼q\left(x_{t}^{i}|x_{t-1}^{i},Z_{t}\right)$$ (3)

The sample set *S*_*t*,1_ is evolved from *S*_*t*−1,1_ with their samples weights according to the target’s motion model and their observation *Z*_*t*_. In this way, at each frame, enough samples are generated at the target region, which will facilitate the optimization of the model. Samples in *S*_*t*,2_ are generated as:

$$x_{t}^{j}∼G\left(\mu_{t},\sigma_{t}\right)$$ (4)

where, *μ*_*t*_ and *σ*_*t*_ are updated at each frame. Each sample in *S*_*t*,2_ is assigned with equal weights. Thus, the samples in *S*_*t*,1_ and *S*_*t*,2_ form the sample set $S_{t}={\left\{\omega_{t}^{i},x_{t}^{i}\right\}}_{i=1}^{n}$.

$$\mu_{t}=s_{t}\times \mu_{0},\sigma_{t}=\sqrt{s_{t}}\times \sigma_{0}$$ (5)

where *s*_*t*_ is the scale change of the target calculated according to ${\hat{X}}_{t-1}$ in the tracking framework.

The samples are generated from prior and current frames, which provides a guarantee for robustness. If all the samples are generated from the previous frame, the samples will concentrate on the better ones, and the accumulated error will loom large. A number of random samples are generated to add new randomness to the sample set.

### Observation

For a specific tracking problem, suppose that the appearance model *M* is integrated by multiple features $O={\left\{o_{i}\right\}}_{i=1}^{m}$, in which the number of features *m* may be fixed (specified features) or adaptive (adapted by an online selection scheme) for different integration models. At each time *t*, the adaptive integration model is defined as

$$M_{t}=F\left(V_{t},O_{t}\right),$$ (6)

where, *O*_*t*_ is the employed feature set, and $V_{t}={\left\{v_{t}^{i}\right\}}_{i=1}^{k}$ is the parameter set. For the models that can be parameterized, the above model *M*_*t*_ is suitable. Employing the integration model to observe the samples *S*_*t*_, likelihood values $D_{t}={\left\{d_{t}^{i}\right\}}_{i=1}^{n}$ are calculated using a proper similarity measure such as

$$D_{t}=Dis\left(M_{t},M\right).$$ (7)

where, *M* is the template of the target. In our experiments, the Bhattacharyya distance is employed to measure the similarity. The sample’s weight is a function value for $d_{t}^{i}$ as $\omega_{t}^{i}=\phi \left(d_{t}^{i}\right)\propto e^{-{d_{t}^{i}}^{2}/\sigma^{2}}$. The likelihood value maps object/background distribution into larger values for samples distinctive to the object and smaller values for samples associated with the background; samples shared by both object and background tend toward medium values.

In our experiments, the target template *M* is updated with the tracking going forward to realize adaptiveness.

$$M=\left\{\begin{array}{cc} M, & D_{t}>T\\ \left(1-\lambda \right)M+\lambda M_{t}, & otherwise \end{array}\right.$$ (8)

where *M*_*t*_ is the tracked region at *i*^*th*^ frame. If the tracking is reliable, the template will be updated; otherwise, it is kept invariable. In our experiments, the parameters *T* and *λ* are set to 0.5 and 0.1, respectively.

### Objective Function

In sum, at each time step, a multi-cue integration model *M*_*t*_ is employed to observe *n* evolved samples, and we got the samples and their weights $S_{t}={\left\{\omega_{t}^{i},x_{t}^{i}\right\}}_{i=1}^{n}$. Now, we want to optimize the integration model *M*_*t*_ to provide a good solution so that it is possible to discriminate the object from its background. Given the knowledge of samples and the prior tracking results, our goal is to build an optimal model with parameters *V*_*t*_. This can be viewed as an optimization problem. The challenge is how to describe “optimize”, that is, how to define the objective function for an optimization algorithm.

**Fig 2:**
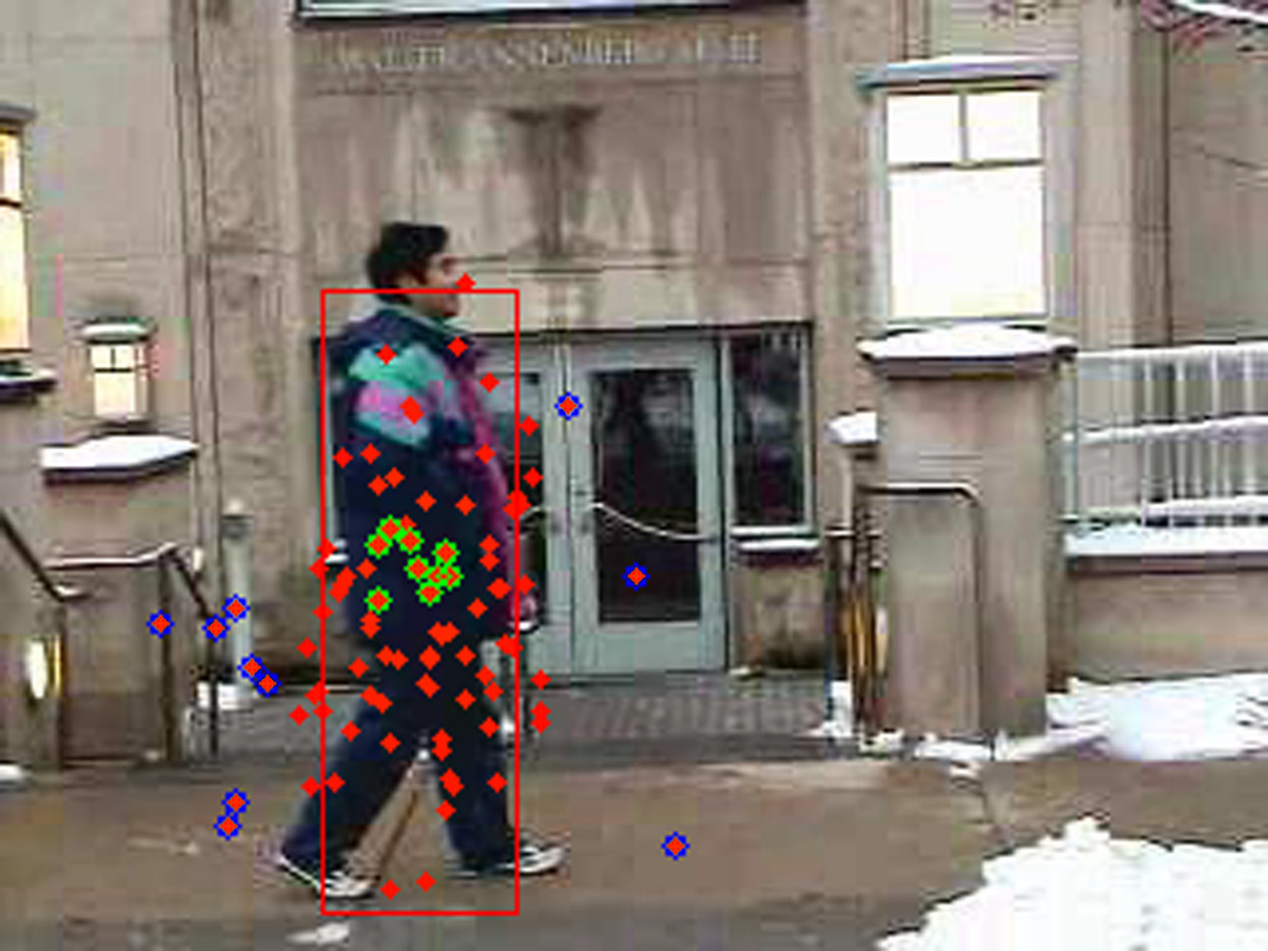
The circled green and blue points are the positive and negative sample sub-sets selected from all the samples (all the points) by a specific appearance model.

As stated previously, our hypothesis is that the features that best discriminate the object from its background are also best for tracking the target. A number of samples have been generated to approximate the object and background pixel distribution. As shown in Fig 1, if a sufficient discriminative model is employed, the weights of these observed samples will show an approximate unimodal distribution like a Gaussian distribution. If a good model is employed, the projection of these samples in the feature space should show a large margin between the two classes. Margin theory has been a hot topic in the machine learning field in the past two decades, until Gao [19] proposed the large margin theory. In his theory, the traditional goal of optimizing the minimum margin algorithm (to maximize the *h*_*min*_ margin which is the minimum distance between two classes) is extended to optimizing not only the minimum margin, but also the margin mean and the margin distribution. Drawing upon his theory, we define the optimization problem by the statistical analysis of these projections in the feature space. Our goal is not to optimize the classification margin but the best positive and negative sample sets selected by the specific integration model, as shown in Fig 2.

Based on the above analysis, we rank the samples by their weights, and extract two sample sets *S*_*o*_ and *S*_*b*_. *S*_*o*_ includes the top *n*_*o*_ samples with higher weights, *n*_*o*_ = *λ*_*o*_ × *n*. They are deemed object pixels with high probabilities. *S*_*b*_ includes *n*_*b*_ samples with lower weights, *n*_*b*_ = *λ*_*b*_ × *n*. They are deemed background pixels with high probabilities. *λ*_*o*_ and *λ*_*b*_ are the experience percentages. Then, a variance like value is computed:

$$val_{t}=\frac{1}{n_{b}}\Sigma_{i=1}^{n_{b}}\left|x_{t}^{i}-\mu_{t}^{o}\right|$$ (9)

where $\mu_{t}^{o}$ is the average value of samples in *S*_*o*_. This value describes the average distance from the samples in the background class to the target center, and it can be viewed as the distance between two classes. In addition, we also compute two variance values as the following:

$$var_{t}^{o}=\frac{1}{n_{o}}\Sigma_{i=1}^{n_{o}}\left|x_{t}^{i}-\mu_{t}^{o}\right|$$ (10)

$$var_{t}^{b}=\frac{1}{n_{b}}\Sigma_{i=1}^{n_{b}}\left|x_{t}^{i}-\mu_{t}^{b}\right|$$ (11)

These two values give a description of the distance within a class.

If a good model is built, the similarity difference between two classes should be as larger as possible, so another value *diff*_*t*_ is calculated:

$$diff_{t}=\left|\frac{1}{n_{o}}\Sigma_{i=1}^{n_{o}}d_{t}^{i}-\frac{1}{n_{b}}\Sigma_{i=1}^{n_{b}}d_{t}^{i}\right|$$ (12)

where, $d_{t}^{i}$ is the similarity of a specific sample.

Based on the above four values, we define an objective function:

$$f\left(V_{t}\right)=\left(var_{t}^{o}var_{t}^{b}\right)/\left(val_{t}diff_{t}\right)$$ (13)

**Fig 3:**
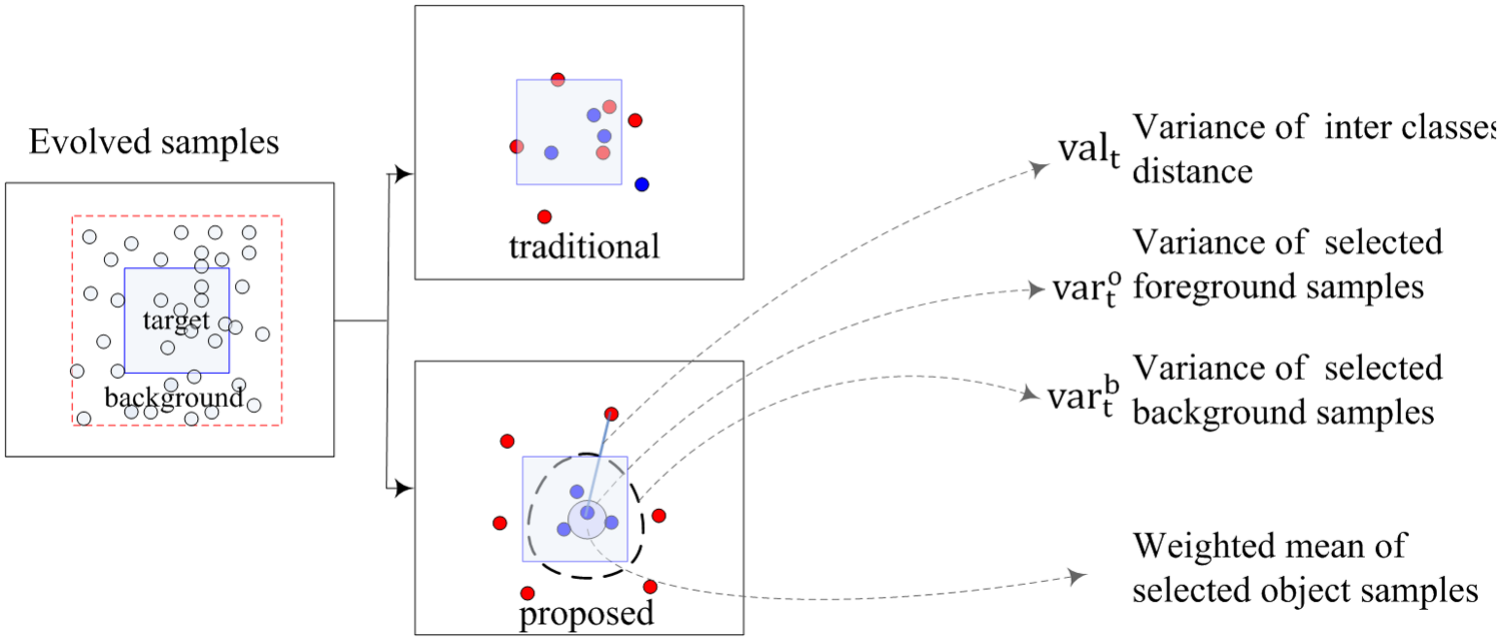
The blue and red points represents the samples from target and background regions, and two different sets are selected by the traditional (upper) and proposed (lower) models. In the previous classification methods, the classification margin is defined by maximizing the minimum interclass distance (in this figure, the distance between the red and blue points). In the definition of optimal model, the sample distribution ($var_{t}^{o}$, $var_{t}^{b}$ and *val*_*t*_) is considered. In comparison, the target and background sample sets selected by the proposed optimal model are of better discrimination, as shown in this figure.

In this definition, our goal is to optimize the samples, defining the margin between object and background. The sample set is selected according to their weights evaluated by the specific integration model. For a specific selected sample set, our goal is to minimize the within-class variance and maximize the between-class distance. Unlike previous classification methods, as shown in Fig 3, the definitions of within- and between- class distances are associated with the sample distribution. The proposed model also shows the importance of margin distribution in defining the classification margin.

At each time step, the target’s appearance model is built by solving the following global optimization problem, defined as finding the parameter vector *V*_*t*_ that minimizes an objective function *f*(*V*_*t*_):

$$Minf\left(V_{t}\right):V_{t}=\left(v_{1},v_{2},\ldots ,v_{i},\ldots ,v_{n}\right)\in R^{n}$$ (14)

which is constrained by the following inequalities and/or equalities:

*l*_*i*_ ≤ *v*_*i*_ ≤ *u*_*i*_, *i* = 1,…,*n*

subject to:

*g*_*j*_(*V*_*t*_)≤0, *for* *j* = 1,…,*p*

*h*_*j*_(*V*_*t*_) = 0, *for* *j* = *p*+1,…,*q*.

*l*_*i*_ and *u*_*i*_ are the lower and upper bound of specific parameters, and *p* and *q*−*p* are the number of the constraint functions *g*_*j*_ and *h*_*j*_, respectively. *f*(*V*_*t*_) is defined on a search space, which is an n-dimensional rectangle in $R^{n}$. This problem is classified into two classes, constrained and unconstrained optimization problems. Typically, the optimization of the appearance model is a constrained optimization problem, and the constraint is defined for specific models. For the model without a constraint, *p* = 0 and *q* = 0. Global optimization is a key problem in applied mathematics, and there are many algorithms that have good performance.

### Optimal adaptive multi-cue integration framework

The proposed optimized integration model is suitable for different feature sets and integration models. In this section, we outline the optimal adaptive framework for any possible extension as follows.

Given a video stream and an initial state *X*_0_ of the interest target, at each time step the model is updated in the following framework.

**Initialization:**

Generate *n* samples $S_{0}={\left\{\omega_{0}^{i},x_{0}^{i}\right\}}_{i=1}^{n}$ with *S*_0_ ∼ *G*(*μ*_0_,*σ*_0_).

**At time t:**

**step1.** Obtain new samples $S_{t}={\left\{\omega_{t}^{i},x_{t}^{i}\right\}}_{i=1}^{n}$ according to formula (2), and produce a solution $V_{t}=\left(v_{1},v_{2},\ldots ,v_{i},\ldots ,v_{n}\right)\in R^{n}$.
**step2.** Perform the following iteration until the termination condition is fulfilled:
  1. observe all the samples *S*_*t*_ by appearance model *M*_*t*_ defined by *V*_*t*_ and feature set *O*_*t*_, and update their weights;
  2. calculate the value of objective function *f*(*V*_*t*_);
  3. employ the global optimization algorithm to optimize *f*(*V*_*t*_) with the parameters constraint.

When the above steps are executed on a given video frame, an optimized solution *V*_*t*_ is obtained, which is the best parameters for the defined model at each frame.

## Tracking

**Fig 4:**
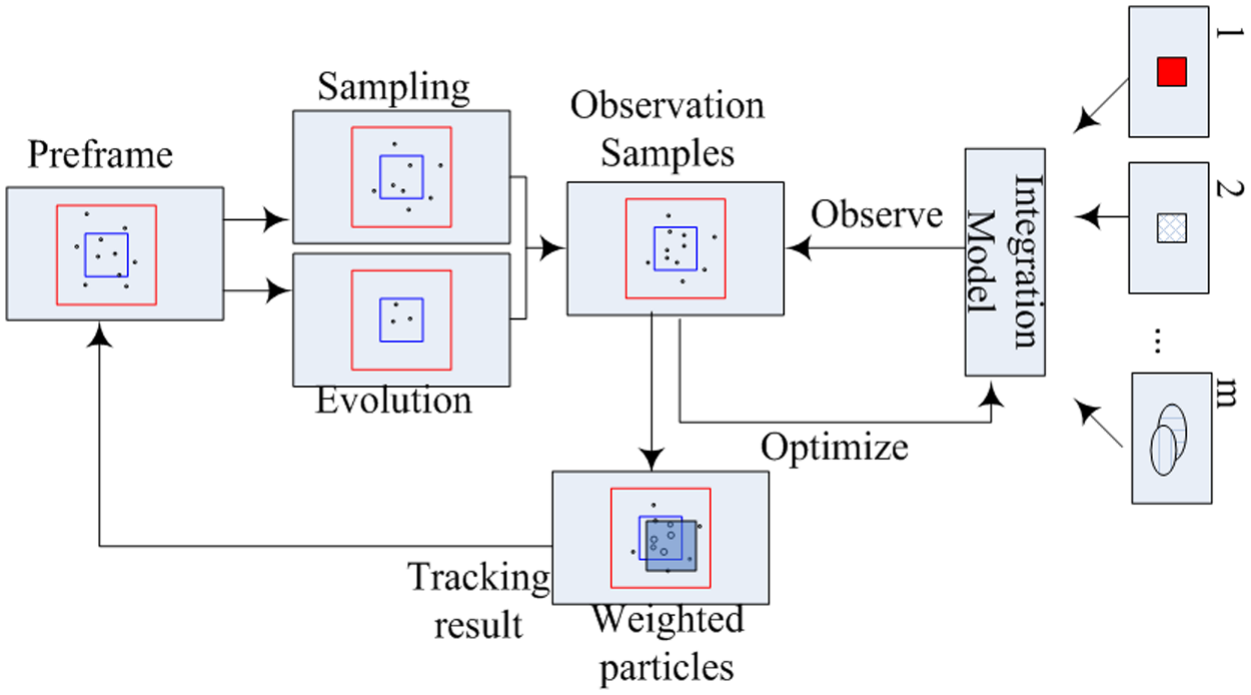
The optimized integration model is embedded in the particle filter framework.

The above optimized feature model framework is embedded in a particle filter (PF) as shown in Fig 4 for the field test. The object and background pixels are partly sampled from the previous frame and partly updated randomly, given the prior knowledge of the previous state of the tracked object and weighted samples. In the PF framework, particles are similar to the samples stated above; for purposes of efficiency, we reuse the particles generated by PF to substitute the samples evolved from the previous frame.

In the particle filter, represent *X*_*t*_ as the target’s state *X*_*t*_, and *Z*_*t*_ as observation at time *t*. On the assumption that the employed *m* cues are orthogonal, the observation model can be written as $Z_{t}=\left(Z_{t}^{1},Z_{t}^{2},\ldots ,Z_{t}^{m}\right)$, and observation likelihood *P*(*Z*_*t*_|*X*_*t*_) is the multi-cue joint similarity.

$$P\left(Z_{t}|X_{t}\right)=\prod_{i=1}^{m}P\left(Z_{t}^{i}|X_{t}\right)$$ (15)

The similarity for each cue is usually represented as a function for distance:

$$P\left(Z_{t}^{i}|X_{t}\right)=\kappa_{t}\left(Z_{t}^{i},T_{i}\right)\propto e^{-d_{i}^{2}\left(Z_{t}^{i},T_{i}\right)/\sigma^{2}}$$ (16)

in which *T*_*i*_ is the template for cue *i* and $d_{i}^{2}\left(Z_{t}^{i},T_{i}\right)$ is the distance between observation $Z_{t}^{i}$ and template *T*_*i*_. Substitute formulas (16) to (15), and *P*(*Z*_*t*_|*X*_*t*_) becomes

$$P\left(Z_{t}|X_{t}\right)=e^{\frac{-\Sigma_{i=1}^{m}1/md_{i}^{2}\left(Z_{t}^{i},T_{i}\right)}{\sigma^{2}}}$$ (17)

Each cue is assigned equal importance. In real tracking conditions, cues have different discriminate ability. More importantly, the model parameters (weights) should be adapted to the condition changes. Therefore, an adaptive multi-cue integration model is represented as the following:

$$P\left(Z_{t}|X_{t}\right)=e^{\frac{-\Sigma_{i=1}^{m}\pi_{t}^{i}d_{i}^{2}\left(Z_{t}^{i},T_{i}\right)}{\sigma^{2}}}$$ (18)

To construct an optimized description using the employed model, a global optimization problem as stated in Eq (13) should be resolved, where the parameters of the model are $V_{t}={\left\{\pi_{t}^{i}\right\}}_{i=1}^{m}$, and $0\leq \pi_{t}^{i}\leq 1$, with the constraint that $\Sigma_{i=1}^{m}\pi_{t}^{i}=1$.

The optimization method is selected according to the defined objective function *f*(*V*_*t*_). If the parameter space is of small size, a traversal in the solution space is of permitted complexity. In addition, if the solution space is of large scale, a certain randomized algorithm like artificial bee colony(ABC) [20] is a good option.

## Experiments

We tested our optimization model on several challenging video sequences. Representative videos have been downloaded from the open video data-sets on the home-page [21] of the paper [22] (which are also available from our web-site with URL: http://ai.nenu.edu.cn/wangyr/OAMVT/OAMVT.htm). In our experiments, the tracking challenges includes complex background(the video “bicycle”, where the man on the bicycle is the target of interest), occlusion(the video “faceocc”, where a woman’s face is frequently occluded partially and totally), target structural variance(the video “skating2”, where a skater is dancing with another skater, and she continuously changes her postures), and abrupt motion(the video “Animal”, where a deer is running in a river, and frequent abrupt motions are shown in the frame), and the target angle changes(the video “girl”, where a girl changes her appearance by shaking her head or turning-around). Overall, the tested videos can be classified into two kinds of challenging conditions: complex scenes and the target’s self changes.

The goal of this work is to demonstrate that using optimization results in a more robust and stable tracker. For this reason, all the parameters for specific features are fixed for all the experiments, and only the integration parameters are optimized. In principle, a wide range of features can be used for tracking, including color, texture, shape, and motion. In this work, we tested the proposed method by representing the target appearance using two types of feature sets. The two models are designed with consideration for different problem scales. One model employed three histogram features (abbreviated as TH) [16], an HSV color histogram, edge histogram, and LBP histogram, that had the property of invariance to changes in scale and rotation. Because the solution space is limited, we used the traversal method to realize the model optimization. The other histograms of color filter bank responses applied to R, G, and B pixel values [15](abbreviated as CFB), and overall, 49 features are employed in the model. With consideration for the problem scale, the artificial bee colony method [20] is employed for optimization. In its implementation, iteration and CPU time are limited in terms of efficiency, and the solution space is decreased by reducing the parameter precision requirement.

To demonstrate the improvement in “adaptiveness”, we compared the optimal model with the adaptive model [16] and fixed model. In the adaptive model [16], the integration parameters (in our experiments, the cue weights) are updated with the tracking reliability. At each frame, each cue weight (weight in Eq (18)) is updated by particle state estimation confidence as:

$$\pi_{t}^{i}=\frac{∥{\hat{X}}_{t}^{i}-{\hat{X}}_{t}∥}{\sum_{i=1}^{m}∥{\hat{X}}_{t}^{i}-{\hat{X}}_{t}∥}$$ (19)

where, ${\hat{X}}_{t}^{i}$ and ${\hat{X}}_{t}$ are the tracking results employing the single cue and integration model, respectively.

To quantitatively evaluate the performance of the proposed optimal model, we compared it with the fixed model and adaptive model without optimization. Two widely accepted evaluation metrics are employed from the tracking literature [23]: the average center location errors (ACLE) and the average bounding box overlap ratio (AOR) [24].

$$CenterError_{i}={∥C_{eval}^{i}-C_{gt}^{i}∥}^{2},ACLE=\frac{1}{M}\Sigma_{i=1}^{M}CenterError_{i}$$ (20)

where *CenterError*_*i*_ is the center error of the *i*^*th*^ frame, and $C_{eval}^{i}$ and $C_{gt}^{i}$ are the tracked and ground-truth object center, respectively.

$$OverlapRate_{i}=\frac{map_{eval}^{i}\cap map_{gt}^{i}}{map_{eval}^{i}\cup map_{gt}^{i}},AOR=\frac{1}{M}\Sigma_{i=1}^{M}OverlapRate_{i}$$ (21)

where *OverlapRate*_*i*_ is the overlap ratio of the *i*^*th*^ frame, and $map_{eval}^{i}$ and $map_{gt}^{i}$ are the tracked and ground-truth bounding box regions. The comparison results are shown in Table 1, and the performance improvement can be seen.

**Table 1:** The tracking performance comparison of the two integration methods on data-sets with varying, sometimes significant changes in object scales. Each entry in the table reports the ACLE and AOR performance.

	CFB						TH					
	fixed		adaptive		Optimal		fixed		adaptive		optimal	
	ACLE	AOR	ACLE	AOR	ACLE	AOR	ACLE	AOR	ACLE	AOR	ACLE	AOR
Faceocc	16.79	0.66	15.81	0.63	14.33	0.74	16.9	0.55	15.8	0.62	14.3	0.68
Skating2	10.35	0.49	7.60	0.49	6.40	0.54	5.97	0.47	5.42	0.57	5.19	0.60
Animal	8.13	0.28	8.10	0.33	7.90	0.36	10.43	0.25	9.87	0.28	8.12	0.34
girl	18.59	0.42	18.50	0.43	17.95	0.44	18.77	0.35	18.58	0.38	18.54	0.4

### Adapting to changeable appearance

**Fig 5:**
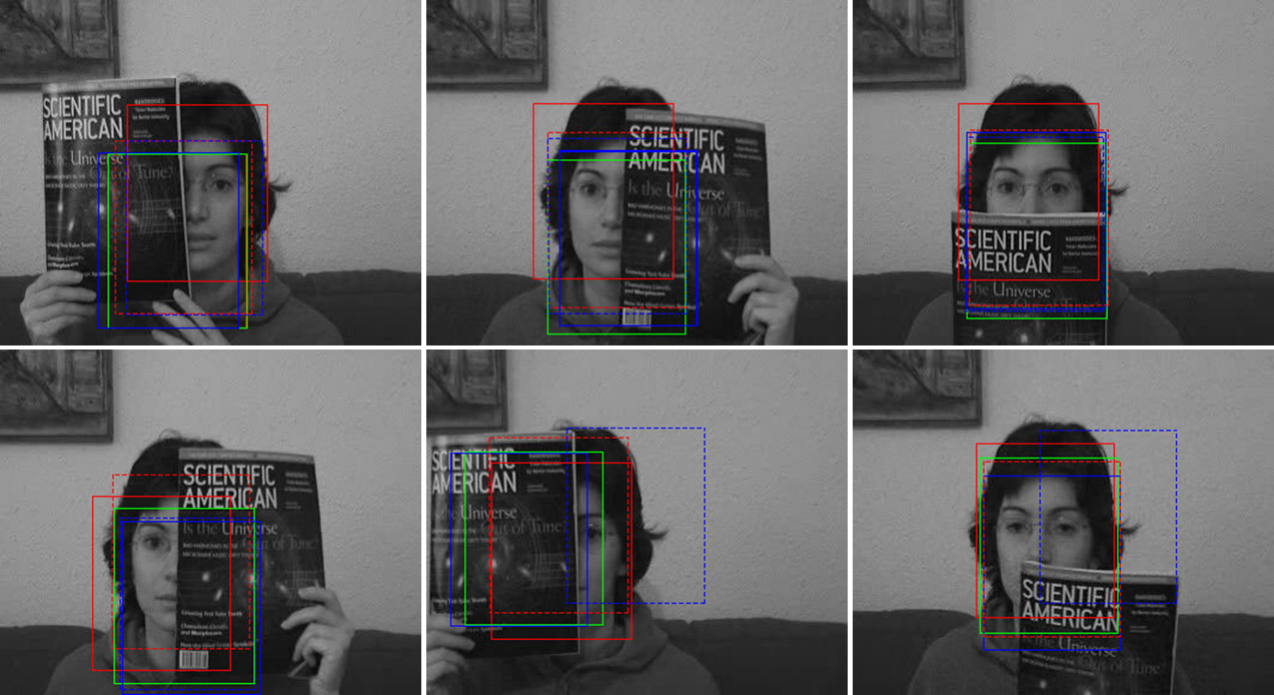
Tracking results of some key frames (♯111, ♯186, ♯301, ♯510, ♯722, ♯885) on video with occlusion, employing TH fixed (blue box), adaptive (green box), optimal models (red box), and 49 CFB models including the fixed one (dashed blue lines) and its optimal version (dashed red lines).

**Fig 6:**
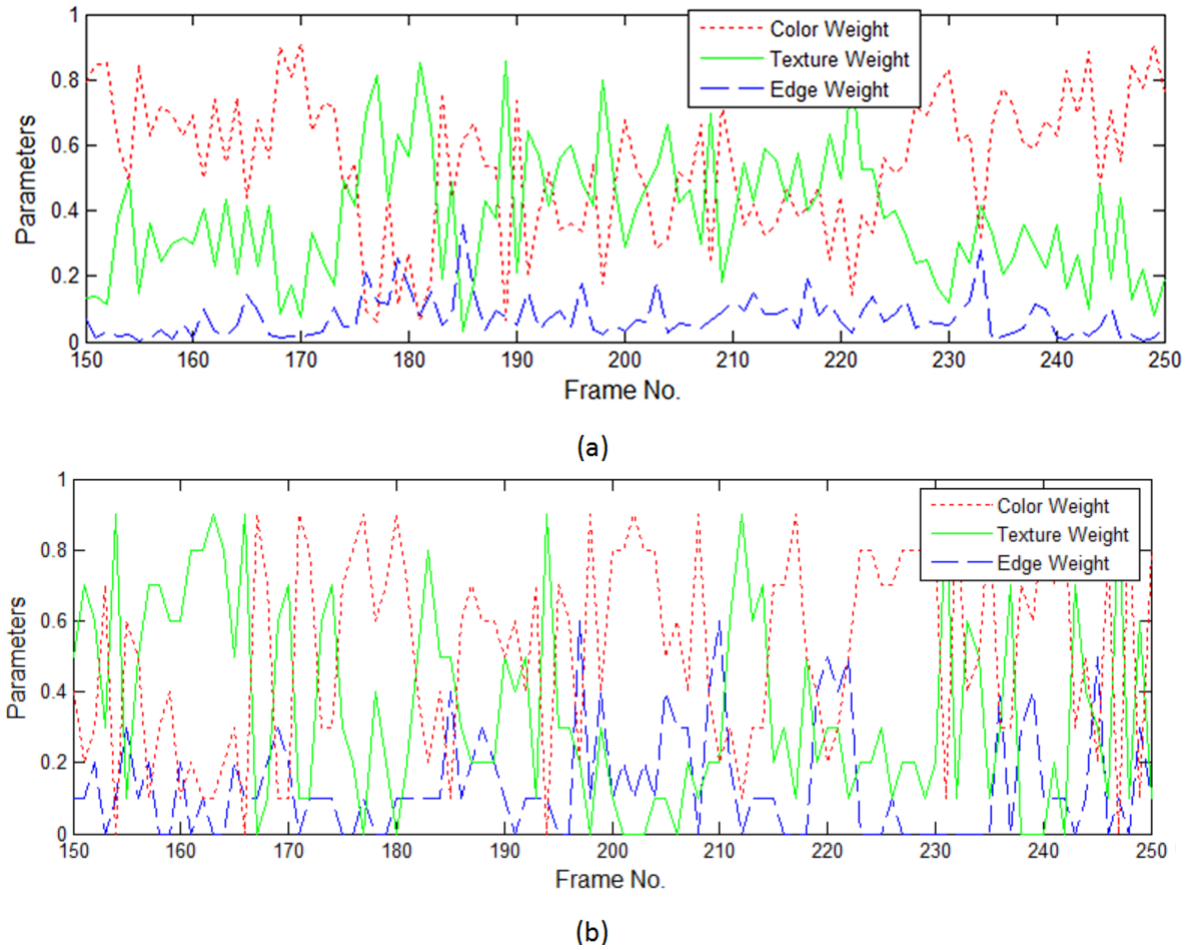
Weight changing curves of TH adaptive models on some selected key frames. (a) Curves for adaptive model, (b) Curves for optimal model, where the change step length is set to be 0.1.

**Fig 7:**
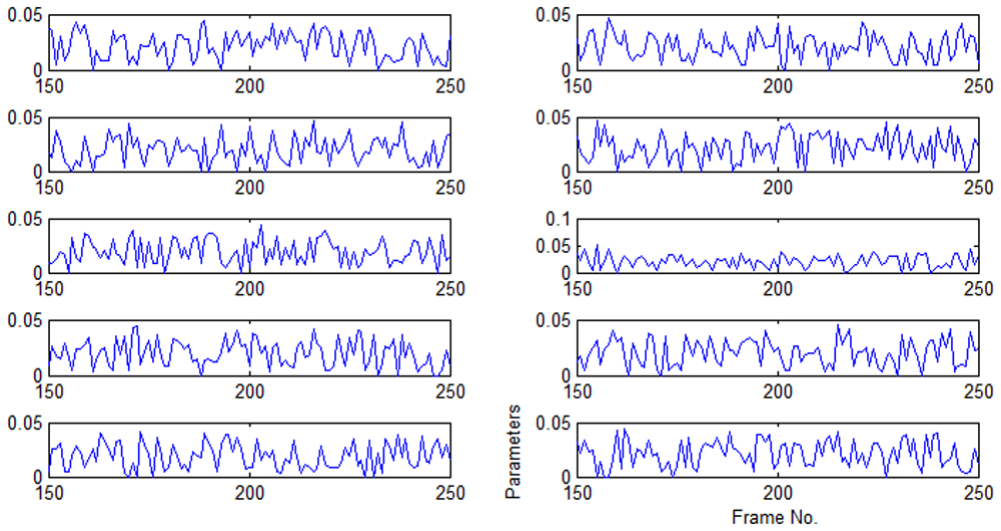
Weights curves for the first ten cues in the CFB model (49 features are employed in observation) show the discriminate ability changes. In this figure, the x axis is the Frame No.(150, 250), and the y label is each cue’s weight value.

The first video is depicted in Fig 5, where a woman’s face undergoes changes to its appearance caused by occlusion from different directions. In some frames of the video, the target face is almost totally occluded. The tracking accuracy of the particle filter framework mainly relies on the performance of the appearance model. Fig 5 gives the results employing the three-cue integration model (TH) and color filter bank (CFB) on some key frames. Accordingly, parameters changing the curves of TH and CFB models are shown in Figs 6 and 7, respectively. In comparison with the tracker of fixed parameters, the TH and CFB adaptive models offer robust tracking and better accuracy. The superior performance of the adaptive models for certain sequences suggests that representing the object with an adaptive model is the right choice for occlusion scenarios. During the entire tracking, both TH adaptive models turn up the ratio of color and texture, and turn down the parameter of the edge, as shown in Fig 6. We witnessed that the adaptive model always performs with less accuracy when the occlusion is large and characterized by abrupt changes. For example, at the frame around ♯200, where almost half of the face is occluded by a book of constrasting color, the two TH adaptive models estimate that the parameter for color should be turned up, but the adaptive model makes a slight change, while the optimal model gives a high ratio to color cue. As a result, the optimal model shows better tracking accuracy. As for the CFB models, the weight curves of the first ten cues (Fig 7) show that discriminant ability changes in different conditions, and the optimized weighted integration shows excellent ability in handling occlusion. On average, less than one millisecond (ms) is required for the TH model on parameter optimization. As for the problem of large scale, ABC is employed for optimization, and an additional 31 ms is required on average. In addition, the code could be simplified for more efficiency.

**Fig 8:**
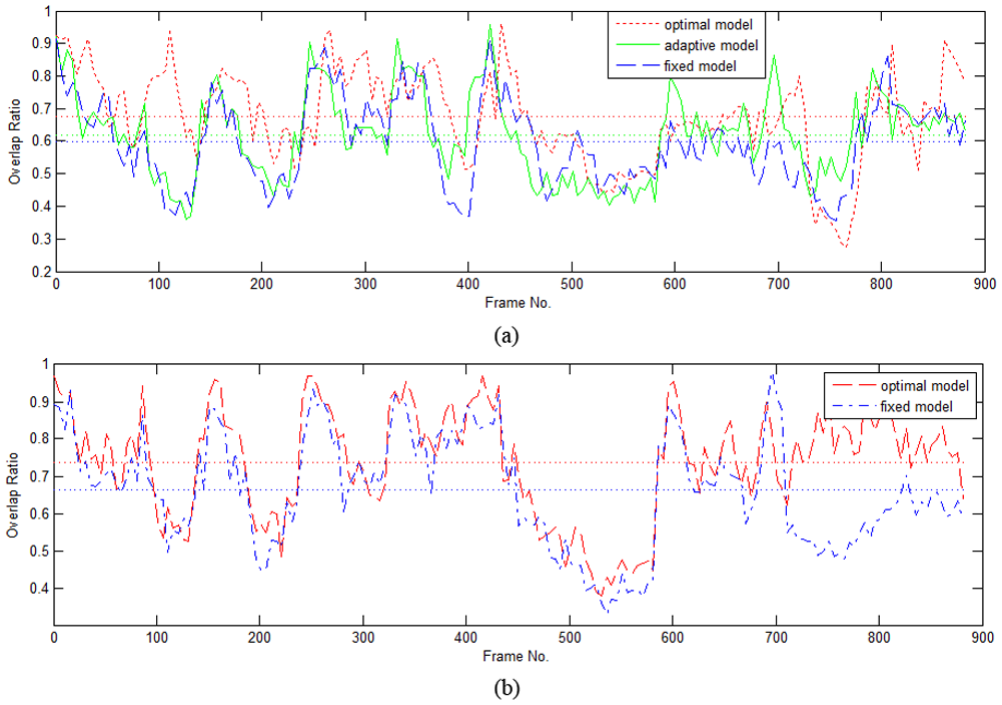
The overlap ratio from the tracking results with the TH optimal model (a) and CFB optimal model (b) for the ground truth data, where, the optimal model shows excellent improvement.

As shown in Fig 8, the overlap ratio at each frame is calculated for the whole video. For both multi-cue models, the bounding box overlap ratio of the optimal model shows dominant performance, especially when there are significant changes to appearance, e.g, around ♯110, ♯710. The goal of our experiment is to test the efficiency of the parameter optimization method with regard to model adaptability. Although the overall rate is only around 0.7, if a more accurate tracking algorithm is employed, like Adaboost-based classification, the tracking accuracy can be improved greatly.

### Adapting to scene background

**Fig 9:**
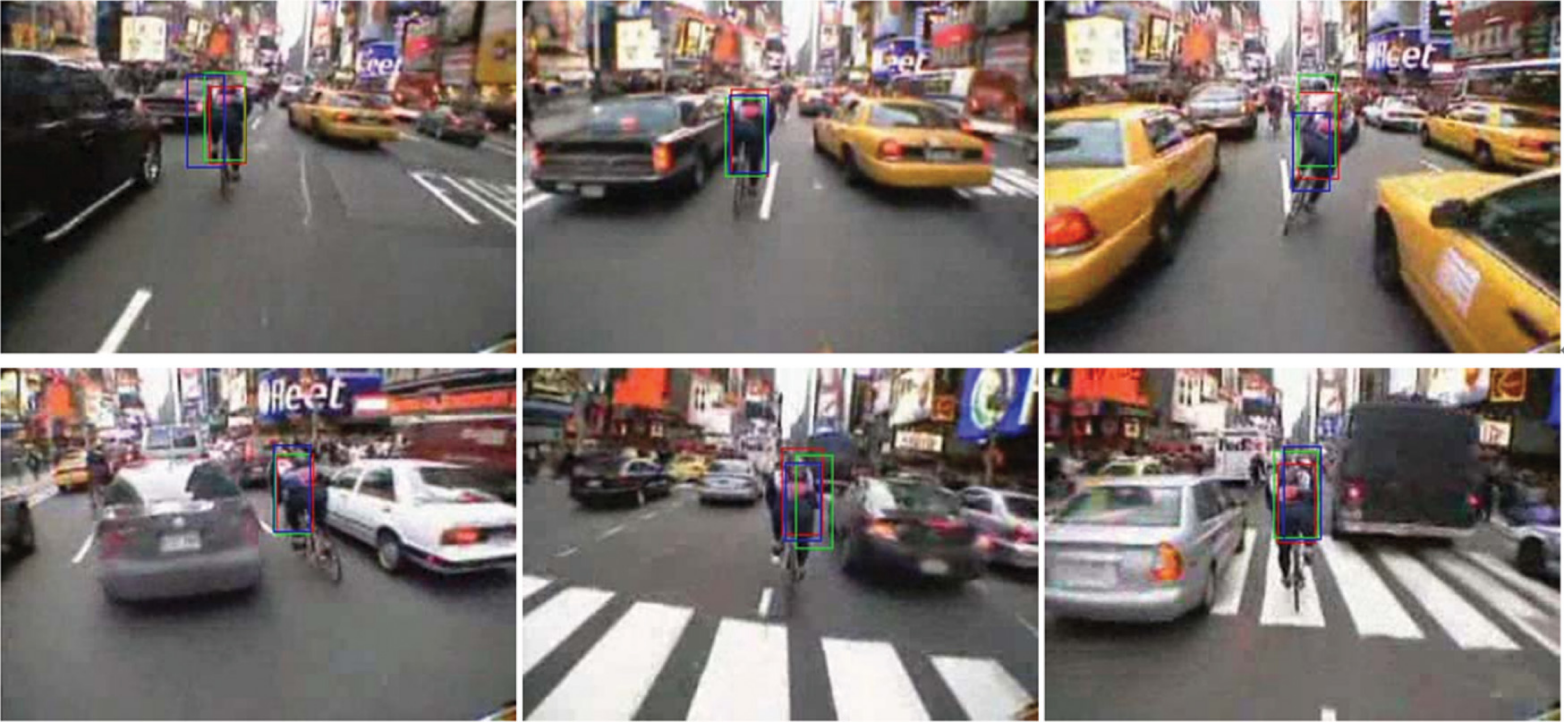
Tracking results of some key frames (♯68, ♯90, ♯127, ♯165, ♯263, ♯300) on video with a changeable complex background, employing fixed model (blue box), adaptive model (green box) and optimal model (red box) for the TH method.

**Fig 10:**
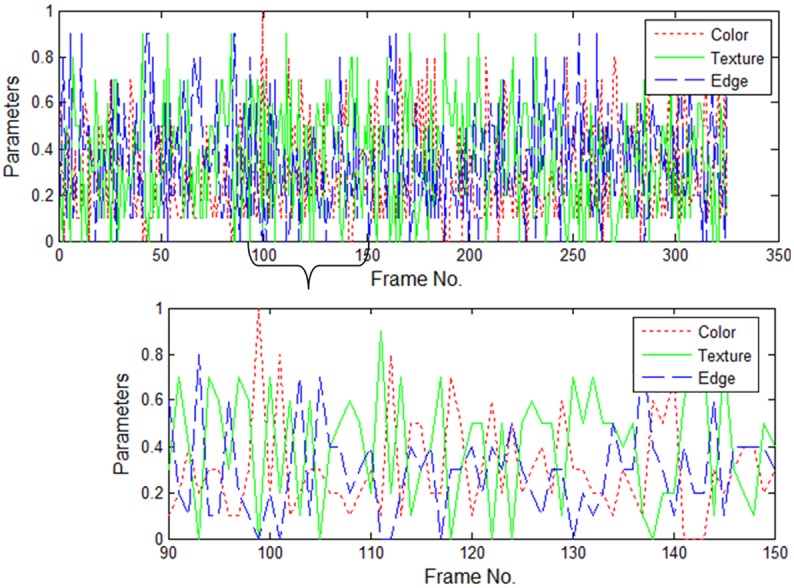
Parameters adapting to the scene background of the TH optimal model. The upper plot is for frames ♯0–♯350, and the lower one is a zoomed in version of frames ♯90–♯150 for clarity.

Fig 9 shows the tracking results for a video with a changeable background. As shown in the figures, a man rides on a street with complex scenes that include vehicles, drivers, and streets, and the background undergoes many changes. Complexity at this level poses challenges to appearance modeling methods because it is hard to build a robust model that is able to distinguish the target from its background. For this video, we tested the fixed, adaptive and optimal models, and they are all able to realize stable tracking. Their successes rely on the employment of integration features with sufficient discriminative ability. However, when their tracking details are compared, the optimal model-based tracking shows superior accuracy. The overall ACLE are 14.6520, 14.1925, and 13.4184 pixels, respectively. Fig 10 provides the model parameters employing TH models. As shown in the figure, at certain key points in the video, the discriminative ability of a specific cue undergoes extensive changes. In such a situation, the fixed model provides cues with unchangeable weight; as a result, tracking accuracy is influenced. For example, around frame ♯100 ∼ ♯150, the target abruptly changes its pose, and the appearance also undergoes many changes. The optimal model continues to adapt parameters, to differentiate the target from the background, thus obtaining greater accuracy.

With regard to scene changes, it is hard for a model to discriminate the target from the background, because the margin between the two classes is constantly undergoing change. Fixed models fail at building a robust margin. In comparison, adaptive models realize robust tracking with less accuracy than the optimal model.

## Discussion

This paper proposed an optimal appearance model, by introducing optimization algorithms in a multi-cue integrating procedure. In the algorithm test period, a particle filter framework was employed due to the requirement of efficiency and non-liner movement in real applications. In addition, comparison with a fixed parameter model and adaptive model was performed to demonstrate the efficiency in robust modeling. The tracking accuracy in the tested system is limited by the accuracy of the particle filter. Currently, the boost-based tracking and detection method is one of the main approaches in visual tracking due to its accuracy. If the proposed optimal model is introduced into the popular boost-based detection method, the accuracy will be much improved; this is the focus of our future work. In addition, a feature database can be built, and our multi-cue integration model can choose discriminative features according to the optimization rule to realize a more robust model.
